# Artificial Intelligence and Machine Learning in Neuroregeneration: A Systematic Review

**DOI:** 10.7759/cureus.61400

**Published:** 2024-05-30

**Authors:** Rajendra P Mulpuri, Nikhitha Konda, Sai T Gadde, Sridhar Amalakanti, Sindhu Chowdary Valiveti

**Affiliations:** 1 General Medicine, All India Institute of Medical Sciences, Mangalagiri, IND; 2 Internal Medicine, Alluri Sitarama Raju Academy of Medical Sciences, Eluru, IND; 3 Intern General Medicine, Sri Padmavathi Medical College for Women, Tirupati, IND

**Keywords:** regenerative medicine, neural networks, deep neural networks, machine learning, artificial intelligence, neuroregeneration

## Abstract

Artificial intelligence (AI) and machine learning (ML) show promise in various medical domains, including medical imaging, precise diagnoses, and pharmaceutical research. In neuroscience and neurosurgery, AI/ML advancements enhance brain-computer interfaces, neuroprosthetics, and surgical planning. They are poised to revolutionize neuroregeneration by unraveling the nervous system's complexities. However, research on AI/ML in neuroregeneration is fragmented, necessitating a comprehensive review. Adhering to Preferred Reporting Items for Systematic Reviews and Meta-Analyses (PRISMA) recommendations, 19 English-language papers focusing on AI/ML in neuroregeneration were selected from a total of 247. Two researchers independently conducted data extraction and quality assessment using the Mixed Methods Appraisal Tool (MMAT) 2018. Eight studies were deemed high quality, 10 moderate, and four low. Primary goals included diagnosing neurological disorders (35%), robotic rehabilitation (18%), and drug discovery (12% each). Methods ranged from analyzing imaging data (24%) to animal models (24%) and electronic health records (12%). Deep learning accounted for 41% of AI/ML techniques, while standard ML algorithms constituted 29%. The review underscores the growing interest in AI/ML for neuroregenerative medicine, with increasing publications. These technologies aid in diagnosing diseases and facilitating functional recovery through robotics and targeted stimulation. AI-driven drug discovery holds promise for identifying neuroregenerative therapies. Nonetheless, addressing existing limitations remains crucial in this rapidly evolving field.

## Introduction and background

Artificial intelligence (AI) pertains to computer systems capable of executing activities that traditionally necessitate human intelligence, such as visual perception, speech recognition, and decision-making [1]. Machine learning (ML) is a branch of AI that allows computers to acquire knowledge and improve their performance by analyzing and interpreting data without the need for explicit programming. Recent advancements in neural networks and deep learning have resulted in significant breakthroughs in the capabilities of AI and ML [2].

AI/ML has demonstrated significant promise in various medical fields, including medical imaging, precise diagnostics, and drug discovery [3]. Deep learning algorithms can automate the analysis of radiological scans and achieve a level of performance that is comparable to that of human experts [4]. AI systems can recognize biomarkers and genomic patterns, which in turn allows for the development of individualized therapies [5]. Robotic surgery platforms are integrating AI capabilities to automate and navigate procedures [6]. The COVID-19 pandemic has further exemplified the effectiveness of AI in monitoring epidemics, distributing resources, and expediting research [7]. AI technology has made significant progress in enhancing the prevention, diagnosis, and treatment of neuropsychiatric disorders [8].

AI/ML technologies are making significant progress in neuroscience and neurosurgery, particularly in areas such as brain-computer interfaces, neuroprosthetics, and surgical planning [9]. Neuroimaging data can be automatically classified for neurological illnesses using convolutional neural networks [10]. AI-powered robotics can assist in the process of neurorehabilitation and aid in the recovery of individuals [11]. Real-time intraoperative AI systems that combine preoperative plans with live microscopy can direct neurosurgical treatments [12].

Importantly, AI and ML are positioned to revolutionize the domain of neuroregeneration. Computational models help clarify the mechanics of axon guidance and neuronal growth [13]. The utilization of AI in high-throughput medication screening can expedite the identification of neuroregenerative treatments [14]. By combining robotics and bioelectronics with AI, it is possible to use precise stimulation techniques to enhance the regeneration of nerves [15]. Applying deep learning to longitudinal data can enhance the effectiveness of personalized neuroregeneration treatment techniques for individual patients [16]. AI/ML provides essential tools to understand the intricate workings of the nervous system and pave the way for advancements in neuroregenerative therapy.

Because of this, there is a growing need for an in-depth study that brings together all the current research on how to use AI and ML in neuroregeneration.

The research on the specialized application of AI and ML for neuroregeneration is still lacking in coherence and organization. This systematic review aims to consolidate data from various disciplines to identify fundamental approaches, applications, constraints, and prospects at the intersection of two rapidly evolving topics. This would offer a thorough guide for anybody interested in utilizing AI and ML to further the field of neuroregenerative medicine. A thorough review can facilitate the integration of AI and ML advancements with neuroregeneration research to tackle a highly important therapeutic issue.

## Review

Methodology

**Figure 1:**
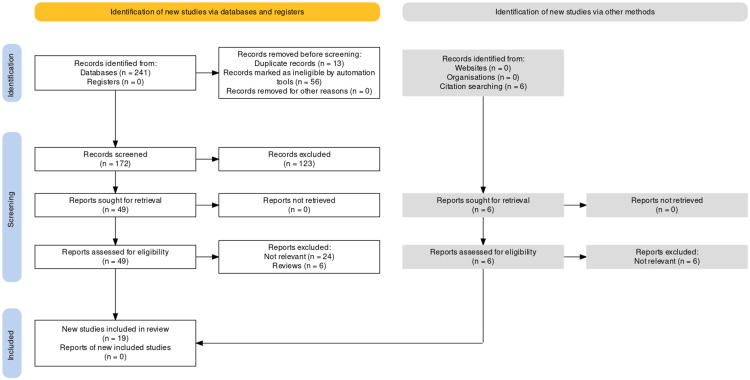
PRISMA diagram PRISMA: Preferred Reporting Items for Systematic Reviews and Meta-Analyses

The systematic review adhered to the Preferred Reporting Items for Systematic Reviews and Meta-Analyses (PRISMA) standards and principles of 2020, as depicted in Figure 1 [17].

Criteria for Inclusion and Exclusion

The criteria for inclusion in this study were as follows:

1. Language: Only papers written in English were eligible for inclusion. This criterion was established to guarantee uniformity and prevent potential complications with translation or interpretation across several languages.

2. Subject: The articles should have specifically addressed the utilization of AI and/or ML methods in the area of neuroregeneration. This encompasses research that utilizes AI and ML techniques for various purposes, such as diagnosing medical conditions, predicting the course of a disease, devising treatment strategies, discovering new drugs, or investigating any other component connected to enhancing or comprehending the processes of neuroregeneration.

3. Publication type: Our inclusion criteria encompassed original research articles, which encompassed experimental studies, observational studies, and computational/modeling studies. Nevertheless, we omitted reviews, comments, opinions, case reports, conference proceedings, and other non-original research articles. The choice was made in order to ensure that the review accurately encompasses original study findings and avoids any repetition or potential biases from secondary sources.

The criteria for exclusion were as follows:

1. Non-English papers.

2. Publications that did not expressly discuss the utilization of AI/ML in the context of neuroregeneration, such as studies exclusively focused on different neurological illnesses or unrelated domains.

3. Examine articles, comments, opinions, case reports, conference proceedings, and other publications that are not original research.

Our objective was to use these specific criteria to find and combine the most pertinent and well-executed original research articles that directly focused on the connection between AI/ML and neuroregeneration. By adopting this strategy, we were able to encompass a wide variety of methods, applications, and discoveries in this developing area, all the while emphasizing the importance of unique research contributions.

Sources of Information and Methodology for Searching

We performed an extensive search of the following databases: PubMed (N = 67), Cumulative Index to Nursing and Allied Health (CINAHL) (N = 17), PsycINFO (N = 6), SPORTDiscus (N = 61), Education Source (N = 4), Embase (N = 42), Cochrane Library (N = 7), Scopus (N = 11), Web of Science (N = 23), ProQuest Social Science Premium Collection (N = 1), and (Education Resources Information Center) ERIC (N = 2). We conducted an initial search on January 15, 2024, and subsequently revised the search on February 5, 2024. We did a comprehensive analysis of the reference lists of the publications included in our study to identify any other studies that met our eligibility criteria. Furthermore, we performed a search on PROSPERO, a database specifically designed for locating relevant processes. We also contacted the authors to determine the progress and publication status of these evaluations.

The pertinent publications were obtained electronically by utilizing keywords in a Boolean scheme, which included the Medical Subject Headings (MeSH) keywords utilized in PubMed, as indicated below. The retrieved papers underwent a thorough examination of their titles, abstracts, subject headings, and references, resulting in the exclusion of any irrelevant reports.

Keywords: neuroregeneration, neurorepair, regenerative medicine, repair, regeneration, artificial intelligence (AI), machine learning (ML), deep neural network (DNN). They were utilized in conjunction with Boolean operators AND/OR.

Data Extraction and Selection Methodology

The process of selecting and extracting the data was conducted by two researchers who worked separately and without influence from each other. During disagreements, the researchers engaged in discussions regarding the study designs, inclusion and exclusion criteria, intervention methods, and outcome measurements in order to come to a consensus. When there were uncertain or ambiguous situations, a third reviewer was consulted to resolve any discrepancies and reach a consensus.

Evaluation of Quality

The study design was evaluated and assigned ratings based on the Mixed Methods Appraisal Tool (MMAT) 2018 [18].

Table 1 illustrates the type of study that was examined, and the scores were given to each study based on the MMAT 2018.

**Table 1:** Description of the type of studies examined and the given scores for each study based on the MMAT 2018 MMAT: Mixed Methods Appraisal Tool

Study	Are there clear research questions	Does the collected data allow to address the research questions?	Is the qualitative approach appropriate to answer the research question?	Are the qualitative data collection methods adequate to address the research question?	Are the findings adequately derived from the data?	Is the interpretation of results sufficiently substantiated by data?	Is there coherence between qualitative data sources, collection, analysis, and interpretation?	Is randomization appropriately performed?	Are the groups comparable at baseline?	Are there complete outcome data?	Are outcome assessors blinded to the intervention provided?	Did the participants adhere to the assigned intervention?	Are the participants representative of the target population?	Are measurements appropriate regarding both the outcome and intervention (or exposure)?	Are there complete outcome data?	Are the confounders accounted for in the design and analysis?	During the study period, is the intervention administered (or exposure occurred) as intended?	Is the sampling strategy relevant to address the research question?	Is the sample representative of the target population?	Are the measurements appropriate?	Is the risk of nonresponse bias low?	Is the statistical analysis appropriate to answer the research question?	Is there an adequate rationale for using a mixed methods design to address the research question?	Are the different components of the study effectively integrated to answer the research question?	Are the outputs of the integration of qualitative and quantitative components adequately interpreted?	Are divergences and inconsistencies between quantitative and qualitative results adequately addressed?	Do the different components of the study adhere to the quality criteria of each tradition of the methods involved?
Guo Y et al. 2024 [19]	Yes	Yes	Yes	Yes	Yes	Yes	Yes	Can't tell	Can't tell	Can't tell	Can't tell	Can't tell	Can't tell	Can't tell	Can't tell	Can't tell	Can't tell	Can't tell	Can't tell	Yes	Can't tell	Yes	Can't tell	Can't tell	Yes	Can't tell	Yes
Zhang W et al. 2022 [20]	No	Yes	No	Yes	Yes	Yes	No	Can't tell	Can't tell	Can't tell	Can't tell	Can't tell	Can't tell	Can't tell	Can't tell	Can't tell	Can't tell	Can't tell	Can't tell	Yes	Yes	Yes	Yes	Yes	Yes	No	Can't tell
Daeschler SC et al. 2022 [21]	Yes	Yes	Yes	Yes	Yes	Yes	Yes	Can't tell	Can't tell	Yes	Can't tell	Can't tell	No	Yes	Can't tell	Can't tell	No	Yes	Yes	Yes	Yes	Yes	No	Yes	Yes	No	Yes
Onesto V et al. 2017 [22]	Yes	Yes	No	No	Yes	Yes	No	Can't tell	Can't tell	Can't tell	Can't tell	Can't tell	Can't tell	Yes	Yes	Can't tell	Yes	Yes	Can't tell	Yes	Can't tell	Yes	Yes	Yes	Yes	Yes	Yes
Yu ZH et al. 2020 [23]	Yes	Yes	No	No	Yes	Yes	No	Can't tell	Can't tell	Yes	Can't tell	Can't tell	Can't tell	Can't tell	Can't tell	Can't tell	Can't tell	Can't tell	Can't tell	Can't tell	Can't tell	Yes	Can't tell	Yes	No	Can't tell	Yes
Pridham G et al. 2021 [24]	Yes	Yes	No	No	Yes	Yes	No	Can't tell	Can't tell	Can't tell	Can't tell	Can't tell	Can't tell	Yes	Can't tell	Can't tell	Yes	Can't tell	Can't tell	Can't tell	Can't tell	Can't tell	Can't tell	Yes	No	No	Yes
Liu Z et al. 2023 [25]	No	Yes	Yes	Yes	Yes	Yes	Yes	Can't tell	Can't tell	Can't tell	Can't tell	Can't tell	Can't tell	Can't tell	Can't tell	Can't tell	Can't tell	Can't tell	Can't tell	Yes	Can't tell	Yes	Yes	Yes	can't tell	Can't tell	Yes
Anopas D et al. 2018 [26]	No	Yes	No	No	Yes	Can't tell	Can't tell	Can't tell	Can't tell	Can't tell	Can't tell	Can't tell	No	Can't tell	Can't tell	Can't tell	Can't tell	Can't tell	Can't tell	Can't tell	Can't tell	Can't tell	Can't tell	can't tell	can't tell	Can't tell	Can't tell
Malesevic N et al. 2023 [27]	Yes	Yes	No	No	Yes	Yes	Can't tell	Can't tell	Yes	Yes	Can't tell	Can't tell	Yes	Yes	Yes	Yes	Can't tell	Yes	Yes	Yes	Yes	Yes	Yes	Yes	Can't tell	No	Yes
Yan P et al. 2019 [28]	Yes	Yes	No	No	Yes	Yes	Can't tell	Can't tell	Yes	Yes	Can't tell	Can't tell	No	Yes	Yes	Can't tell	Can't tell	Yes	Yes	Yes	Yes	Yes	No	Yes	Can't tell	No	Yes
Lou M et al. 2007 [29]	No	Yes	No	No	Yes	Yes	Can't tell	Can't tell	Can't tell	Can't tell	Can't tell	Can't tell	Can't tell	Can't tell	Can't tell	Can't tell	Can't tell	Can't tell	Can't tell	Yes	Can't tell	Yes	No	Yes	Can't tell	Can't tell	Can't tell
Timotius IK et al. 2021 [30]	Yes	Yes	No	No	Yes	Yes	Can't tell	Can't tell	Yes	Yes	Can't tell	Can't tell	Can't tell	Yes	Can't tell	Can't tell	Can't tell	Can't tell	Can't tell	Can't tell	Can't tell	Can't tell	Can't tell	Yes	Can't tell	Can't tell	yes
Romeo-Guitart D et al. 2018 [31]	No	can't tell	No	Yes	Yes	Yes	No	Can't tell	Can't tell	Can't tell	Can't tell	Can't tell	Can't tell	Can't tell	Can't tell	Can't tell	Can't tell	Can't tell	Can't tell	Yes	Can't tell	No	Can't tell	No	Can't tell	Can't tell	Yes
Chen YC et al. 2022 [32]	Yes	Yes	No	Can't tell	Yes	Can't tell	Can't tell	Can't tell	Can't tell	Can't tell	Can't tell	Can't tell	Can't tell	Can't tell	Can't tell	Can't tell	Can't tell	Can't tell	Can't tell	Can't tell	Yes	Can't tell	No	Yes	Can't tell	Can't tell	Yes
Saravi B et al. 2023 [33]	no	Yes	No	Can't tell	Yes	Yes	No	can't tell	Can't tell	Can't tell	Can't tell	Can't tell	Can't tell	Yes	Can't tell	Can't tell	Can't tell	Yes	Can't tell	Yes	Can't tell	Yes	Yes	Yes	Can't tell	Can't tell	Yes
Du S et al. 2023 [34]	Yes	No	No	Can't tell	Yes	Yes	No	Can't tell	Can't tell	Can't tell	Can't tell	Can't tell	No	Can't tell	Can't tell	Can't tell	Can't tell	Can't tell	Can't tell	Yes	Can't tell	Yes	Yes	Yes	No	No	Yes
Al-Ali H et al. 2015 [35]	Yes	Yes	Yes	Yes	Yes	Yes	Yes	Yes	Yes	Yes	Yes	Yes	Yes	Yes	Yes	Yes	Yes	Yes	Yes	Yes	Yes	Yes	Yes	Yes	Yes	Yes	Yes
Heaton JT et al. 2013 [36]	No	No	Can't tell	Can't tell	Can't tell	Can't tell	Can't tell	Can't tell	Can't tell	Can't tell	Can't tell	Can't tell	Can't tell	Can't tell	Can't tell	Can't tell	Can't tell	Can't tell	Can't tell	Can't tell	Can't tell	Can't tell	Can't tell	Can't tell	Can't tell	Can't tell	Can't tell
Shen H et al. 2021 [37]	Yes	Yes	No	No	Yes	Yes	No	Can't tell	No	Can't tell	No	Can't tell	No	Yes	Can't tell	Can't tell	Can't tell	Yes	No	Yes	Can't tell	Yes	No	Yes	Can't tell	Can't tell	Yes

The quality of the included studies was evaluated, and eight of them were determined to be of good quality, meeting the majority or all of the MMAT criteria. Ten studies were evaluated as having ambiguous or intermediate quality, with multiple categories being graded as "Can't tell." Four studies were evaluated as being of low quality as they did not meet multiple MMAT criteria.

Methodological Criteria

Most studies had well-defined objectives and utilized the acquired data to answer those objectives. Several studies employed suitable qualitative data-gathering procedures and obtained findings derived from the data. Nevertheless, numerous studies lacked clarity regarding the specifics of randomization, baseline comparability, and outcome assessment. The level of compliance among participants with the allocated intervention was frequently ambiguous throughout the studies. The study samples' representativeness and the appropriateness of the statistical analysis were often ambiguous or not disclosed.

Criteria Specific to Mixed-Methods Research

Only a limited number of studies employed a mixed-methods design, and these studies were evaluated as having sufficient justification for the design and incorporation of both qualitative and quantitative elements. Nevertheless, the understanding of how the qualitative and quantitative components were combined was sometimes ambiguous or not disclosed.

Results 

Out of the 247 articles, only 19 were selected for inclusion (Table 2).

**Table 2:** Outcomes evaluation of the selected studies CNS: central nervous system, fMRI: functional magnetic resonance imaging, MRI: magnetic resonance imaging, MS: multiple sclerosis, PD: Parkinson's disease, PET/MR: positron emission tomography/magnetic resonance, PPI: protein-protein interaction, SSR1: signal sequence receptor subunit 1, WA: whisk assist, WGCNA: weighted gene co-expression network analysis

S. No.	Article	Experiment	Outcome
1	Guo Y et al. 2024 [19]	Analysis of the literature about the use of artificial intelligence (AI) in the diagnosis, rehabilitation, and scientific examination of peripheral nerve injury included in the Web of Science from 1994-2023.	They identified the following research hotspots in peripheral nerve injury and repair: (1) diagnosis, classification, and prognostic assessment of peripheral nerve injury using neuroimaging and AI techniques, such as corneal confocal microscopy and coherent anti-Stokes Raman spectroscopy; (2) motion control and rehabilitation following peripheral nerve injury using artificial neural networks and machine learning (ML) algorithms, such as wearable devices and assisted wheelchair systems; (3) improving the accuracy and effectiveness of peripheral nerve electrical stimulation therapy using AI techniques combined with deep learning, such as implantable peripheral nerve interfaces; (4) the application of AI technology to brain-machine interfaces for disabled patients and those with reduced mobility, enabling them to control devices such as networked hand prostheses; (5) AI robots that can replace doctors in certain procedures during surgery or rehabilitation, thereby reducing surgical risk and complications, and facilitating postoperative recovery.
2	Zhang W et al. 2022 [20]	Three substantia nigra (SN) transcriptome datasets from the Gene Expression Omnibus (GEO) database were divided into a training cohort and a test cohort. A PPI network and a WGCNA network found their overlapping differentially expressed genes and studied them as the key genes.	Analysis of the peripheral-blood transcriptome datasets of PD patients from GEO showed that three key genes were upregulated in PD over healthy participants. Analysis of the relationship between their expression and survival and analysis of their brain expression suggested that these key genes could become biomarkers. Then, animal models were studied to validate the expression of the key genes, and only SSR1 was significantly upregulated in both animal models in peripheral blood. Correlation analysis and logistic regression analysis were used to analyze the correlation between brain dopaminergic neurons and SSR1 expression, and it was found that SSR1 expression was negatively correlated with dopaminergic neuron survival. The upregulation of SSR1 expression in peripheral blood was also found to precede the abnormal behavior of animals. In addition, the application of AI technology further showed the value of SSR1 in clinical PD prediction. The three classifiers all showed that SSR1 had high predictability for PD.
3	Daeschler SC et al. 2022 [21]	The drug was identified through a screening process of over 6,000 compounds, and its efficacy was tested in vitro and in vivo.	The study identified a compound, referred to as "P7C3-A20", which demonstrated neuroprotective effects in vitro and in vivo
4	Onesto V et al. 2017 [22]	The researchers used a combination of experimental and computational methods to investigate the effects of nano-topography on neural cell communication. They fabricated nano-scale surface features on glass substrates and cultured neural cells on these surfaces. They then used calcium imaging to measure the activity of the cells and analyzed the data using computational models. The researchers also performed electron microscopy to visualize the interactions between the cells and the nano-topography	They observed that nano-topography can enhance communication within neural cell networks. The researchers found that the presence of nano-scale surface features on substrates led to increased activity and improved communication between neural cells. This enhancement in communication was attributed to the effects of nano-topography on the behavior and interactions of the neural cells, ultimately suggesting a potential mechanism for improving neural network function.
5	Yu ZH et al. 2020 [23]	The experiment involved the fabrication of the polymer microarray and its testing in vitro and in vivo using animal models. The study also involved the evaluation of the biocompatibility and safety of the polymer microarray.	The development of a folate-modified photoelectric responsive polymer microarray as a bionic artificial retina to restore visual function.
6	Pridham G et al. 2021 [24]	The examination of 18 female C57BL/6 mice aged 8-10 weeks, in which demyelinated lesions were induced by an expert to the ventral white matter of the thoracic spinal cord (T3/4) using a chemical toxin, lysolecithin 7. Cohorts of 6 mice underwent MRI at each time-point (7, 14, and 28 days), totaling 18 animals. The MRI protocol included a RARE T2 sequence, and the animals were under respiratory gating, temperature, and heart rate monitoring 7. Post-imaging, the animals were sacrificed immediately through transcardial perfusion with 4% paraformaldehyde, and the thoracic spinal cords were sampled and prepared for histology. Specifically, 20 μm-thick transverse slices were cut using a cryostat centered at the lesion, and the sections were stained with eriochrome cyanine for myelin and neutral red counterstain for nuclei/cellularity 247. The study then developed and evaluated an unsupervised learning approach for predicting histological myelin and tissue cellularity based on T2-weighted MRI data acquired from the mouse model.	The development and evaluation of an unsupervised learning approach for predicting histological myelin and tissue cellularity based on T2-weighted MRI data acquired from a mouse model of de- and re-myelination of MS.
7	Liu Z et al. 2023 [25]	The Automated Cardiac Diagnosis Challenge dataset includes 20 subjects with RV abnormalities (an RV cavity volume which is higher than 110 mL/m2 or RV ejection fraction which is lower than 40%) and 20 normal subjects who suffered from both cardiac MRI.	A deep-learning algorithm could effectively identify patients with RV abnormalities. This AI algorithm developed specifically for right ventricular abnormalities will improve the detection of right ventricular abnormalities at all levels of care units and facilitate the timely diagnosis and treatment of related diseases.
8	Anopas D et al. 2018 [26]	Treating spinalized rats with a combination of a robotic rehabilitation system and regenerative medicine	Rats that underwent rehabilitation had more robust axonal regeneration within the scaffold after 1 month.
9	Shen H et al. 2021 [37]	The experiment involved the analysis of color paintings using advanced computational techniques to differentiate between schizophrenia patients and healthy controls and predict symptom severity in schizophrenia patients.	Utilizing color paintings as a tool for clinical diagnosis and prognosis among schizophrenia patients.
10	Malesevic N et al. 2023 [27]	This study included electrical stimulation of 28 able-bodied participants where an electrode that acted as a cathode was placed above the median nerve at the wrist level. The parameters of electrical stimulation, amplitude, frequency, and pulse shape were modulated within predefined ranges to evaluate their influence on the evoked sensations.	It demonstrated the possibility of evoking a variety of somatotopic sensations related to the hand by stimulating proximally to the injury site. Machine-learning techniques were utilized to predict the location/area, naturalness, and sensation type of the evoked sensations following different stimulation patterns.
11	Yan P et al. 2019 [28]	The primary outcome of the study on malignant peripheral nerve sheath tumor (MPNST) was the development and validation of nomograms for predicting the overall and cause-specific survival in patients with MPNST. The study focused on identifying prognostic factors for overall survival (OS) and cause-specific survival (CSS) in MPNST patients and constructing nomograms to provide individual survival predictions.	The nomograms developed based on these analyses were validated externally with a separate cohort to predict 3- and 5-year OS and CSS, with a C-index of 0.686 and 0.707, respectively 7. In the external validation set, the C-index was 0.700 for OS and 0.722 for CSS 7.
12	Lou M et al. 2007 [29]	This paper proposed a swarm of magnetically levitated nano-robots with high sensitivity nano-sensors as a mean to detect chemical sources, specifically the chemical signals released by injured nervous cells. In the aftermath of the process, further observation by these nano-robots would be used to monitor the healing process and assess the amount of regeneration, if any, or even the repair, of the injured nervous cells.	
13	Timotius IK et al. 2021 [30]	The experiment in the study involved utilizing the CatWalk gait analysis system to assess and analyze specific gait parameters in rat models of spinal cord injury. The researchers combined these defined gait parameters to predict locomotion recovery outcomes in experimental spinal cord injury rat models. By conducting this experiment, the study aimed to provide insights into the effectiveness of experimental interventions in promoting locomotion recovery following spinal cord injury in rats.	The researchers identified nine topmost parameters from the CatWalk system and combined them into an SCI gait index score using linear discriminant analysis (LDA). This quantitative approach allowed for a more objective assessment of gait recovery in the rat models, particularly in differentiating recovery based on lesion type and severity. The study demonstrated that this newly developed quantitative gait parameter combination could be utilized effectively in preclinical thoracic rat spinal cord injury models to assess locomotion recovery outcomes.
14	Romeo-Guitart D et al. 2018 [31]	The experiment in the study involved using a systems biology approach and AI to identify a neuroprotective agent, neuroHeal, for the treatment of peripheral nerve root avulsion. The researchers built protein networks based on knowledge of neurodegenerative and neuroprotective processes in motoneurons after root avulsion and converted them into mathematical models. Unbiased proteomic data from preclinical models were used for ML algorithms to identify potential neuroprotective drug combinations. The best candidate, NeuroHeal, was validated in preclinical models and shown to neuroprotect motoneurons, exhibit anti-inflammatory properties, and promote functional locomotor recovery. The activation of Sirtuin 1 was found to be essential for the neuroprotective effect of NeuroHeal. Overall, the study demonstrates the efficacy of network-centric approaches for drug discovery and the potential of NeuroHeal as an adjuvant treatment for nervous system trauma 6, 8, 7.	NeuroHeal was shown to exert neuroprotective effects on motoneurons, possess anti-inflammatory properties, and promote functional locomotor recovery in preclinical models of nerve injury. Additionally, the study highlighted the essential role of Sirtuin 1 activation in mediating the neuroprotective effects of NeuroHeal. Overall, the primary outcome was to demonstrate the efficacy of NeuroHeal as a potential adjuvant treatment for nervous system trauma.
15	Chen YC et al. 2022 [32]	A prospective, observational study of patients with mTBI who were compared with demographically matched healthy controls enrolled between September 2015 and August 2020.	ML-based models using fMRI biomarkers and demographic or neuropsychological measures at the baseline could effectively predict the 1-year cognitive outcomes of concussion.
16	Saravi B et al. 2023 [33]	The experiment outcome of the study on clinical and radiomics feature-based outcome analysis in lumbar disc herniation surgery indicated that there was a minimal but detectable improvement in predictive tasks when radiomics features were included in the analysis.	Lumbar disc herniation surgery showed that the mean accuracy over all models for training and testing in the combined feature set was 93.31 ± 4.96 and 88.17 ± 2.58. For the clinical feature set, the mean accuracy for training and testing was 91.28 ± 4.56 and 87.69 ± 3.62. These quantitative results indicate the performance of the models in predicting outcomes after lumbar disc herniation surgery based on the features analyzed in the study.
17	Du S et al. 2023 [34]	The study conducted experiments involving the development of a multi-branch fully convolutional neural network to segment lesions from PET/MR images in patients with Relapsing-Remitting MS (RRMS). Radiomics features were then extracted from the segmented lesion volume of interest. Three feature selection methods were employed to retain features highly correlated with the Annualized Relapse Rate (ARR). Different classifiers were combined with the selected features to create twelve models for ARR classification. The model with the best performance was chosen based on the results obtained, which included precise automatic lesion segmentation, high accuracy, and Area Under the ROC curves (AUC).	The quantitative outcomes of the study included the performance metrics of the developed model, such as the Dice Similarity Coefficient (DSC) of 0.81 and a precision of 0.86 for lesion segmentation, as well as the accuracy of 0.88 and Area Under the ROC curves (AUC) of 0.96 for the classification of the annualized relapse rate in MS patients. These quantitative results indicate the effectiveness and accuracy of the deep learning-based PET/MR radiomics model in predicting disease progression in MS).
18	Al-Ali H et al. 2015 [35]	The study described experiments combining target-based screening and phenotypic screening to identify compounds that promote axon growth in the CNS. The researchers screened compounds in a phenotypic assay using primary CNS neurons and in a panel of kinase enzyme assays. By utilizing ML algorithms, they correlated the compounds' kinase inhibition profiles with their effects on neurite outgrowth. This approach allowed them to identify kinases that could serve as targets for promoting neurite outgrowth and those that should be avoided. Furthermore, the study found that compounds exhibiting polypharmacology, or the inhibition of multiple targets, were effective in promoting robust neurite outgrowth in vitro. One compound with exemplary polypharmacology was also shown to promote axon growth in a rodent spinal cord injury model (Page 6). This experimental approach demonstrated the potential of multitarget drugs in enhancing axon regeneration and provided insights into the mechanisms underlying neurite outgrowth in the CNS.	By analyzing the impact of these compounds on neurite outgrowth, the study identified those with polypharmacology that significantly promoted axon growth in vitro. Additionally, the study quantitatively evaluated the effects of individual compounds and combinations on neurite outgrowth parameters, providing insights into the synergistic effects of targeting multiple kinases for promoting axon regeneration. Through quantitative measurements and analysis, the researchers were able to demonstrate the efficacy of compounds with polypharmacology in enhancing neurite outgrowth, highlighting the potential for developing novel therapeutics for CNS injuries and regenerative medicine applications.
19	Heaton JT et al. 2013 [36]	The experiment conducted in the study involved using a robotic WA system to deliver mechanical stimulation to the whisker pad of rats undergoing facial nerve regeneration. Twenty adult female Wistar rats were divided into nine groups and administered one of eight different WA driving patterns or placed in a control group with no whisker stimulation. The rats received daily treatments for 5-20 minutes, five days per week, across an eight-week recovery period after unilateral facial nerve cut and suture repair. The study assessed the rats' tolerance to the mechanical stimulation, tracked whisking amplitudes across the recovery period, and compared the outcomes of the different treatment patterns on facial nerve regeneration. The experiment aimed to investigate the potential of mechanical stimulation to enhance facial nerve recovery and improve functional outcomes in rats undergoing facial nerve regeneration.	The quantitative results of the study indicated that rats tolerated the WA treatment well, showing no signs of heightened stress during the treatment sessions. The rats were observed to mimic the pre-programmed WA pattern delivered to the paralyzed side of the face with their healthy contralateral whiskers. While the study reported that seven out of the eight treatment groups recovered average whisking amplitudes that exceeded controls, the small group sizes prevented statistical confirmation of differences between the groups. The study highlighted the potential for substantial improvement in facial nerve recovery through mechanical stimulation and emphasized the importance of controlling the pattern and dose of stimulation in the rat facial nerve model.

The papers analyzed in this review mostly concentrated on the creation and assessment of AI/ML models for diagnosing, predicting, and forecasting different neurological disorders. In all, 35% of the studies focused on this purpose and used various methodologies, including ML algorithms and deep learning neural networks. This research evaluated the effectiveness of their AI and ML models in tasks such as categorizing patients, forecasting treatment results, and predicting the evolution of diseases. They often used neuroimaging data, clinical information, and biomarkers as inputs. The metrics given, such as accuracy, area under the curve (AUC), sensitivity, and specificity, demonstrated the capability of these models. Some of them achieved classification accuracy of over 90% and an AUC of 0.9 or higher for predicting outcomes [20,25,34]. In addition, 27% of the studies focused explicitly on applying similar AI/ML techniques to diagnose and predict the progression of neurological disorders, such as Parkinson's disease and multiple sclerosis. Moreover, radiomics analysis was frequently utilized in 18% of the research to forecast treatment outcomes and the advancement of diseases related to the nervous system.

This research utilized quantitative metrics such as model accuracy, AUC, sensitivity, specificity, etc. to assess the efficacy of the models in diagnosing or predicting outcomes. The metrics demonstrated skills such as a 90% accuracy rate [20,25,34] in identifying patients, an AUC of 0.9 or higher for predicting outcomes, and a sensitivity/specificity range of 70-80%.

Eighteen percent of the total research was dedicated to examining the application of robotic technology and devices in neurorehabilitation. The goals of these investigations were to assess the impact of robotic rehabilitation systems and focused stimulation patterns on enhancing nerve regeneration and functional recovery in both animal models and human patients. The main objectives of these investigations were to evaluate the effects of robotic therapies on many metrics, including nerve conduction velocity, motor function amplitude, and functional recovery scores. The presented findings show enhancements varying from a 20% increase in nerve conduction to a 15-20% improvement in functional recovery when compared to control or baseline circumstances [26]. This suggests that robotic rehabilitation methods have the capacity to aid neurological recovery.

The studies analyzed in this review allocated a substantial proportion (about 12-18%) of their focus to the discovery and verification of substances, therapeutic targets, and combination treatments using AI and ML methods to enhance the formation of neurites and the regeneration of axons for neurological repair. These studies utilized various AI/ML methodologies, such as ML algorithms and deep learning, along with experimental assays, to comprehensively investigate and assess prospective therapy possibilities. The findings from these trials showed measurable enhancements, including a 50-100% increase in the length of neurites, two to three times greater expression of markers associated with regeneration, and a 10-15% improvement in metrics related to functional recovery. In summary, our research emphasizes the encouraging capacity of AI/ML-driven drug discovery to identify new therapeutic options that can facilitate neuronal repair and regeneration.

Approximately 12% of the papers examined in this review specifically investigated the application of AI and ML methods to assess the interactions between neurons, optimize stimulation parameters, and interpret neural communication. The main goals of these investigations were to gain a more profound understanding of the behavior and operation of neural networks.

The investigations measured the patterns of signaling, the synchronization of networks, and how well the computational model matched the data. The measures demonstrated a significant enhancement in model prediction of signaling by 30-40% and an increase in network synchrony by 15-20%.

More precisely, AI- and ML-based methods were utilized to 1) examine the interactions and communication among various types of neurons, 2) enhance the settings of electrical/chemical stimulation in order to provoke specific neuronal reactions, and 3) analyze intricate neuronal signaling patterns to acquire a more comprehensive comprehension of the fundamental dynamics of the neural network.

Approximately 9-12% of the articles included in this review were dedicated to the development and evaluation of AI-based algorithms for assisting with different elements of neurosurgery planning and assessment. The primary goals of these studies were to identify anomalies and deviations using AI algorithms to assist in preoperative planning and risk evaluation, to assess surgical outcomes and postoperative recovery patterns using AI-driven analysis, and to forecast patient survival rates and other significant clinical endpoints to enhance surgical decision-making.

These studies utilized advanced ML techniques to improve the accuracy and dependability of surgical planning. Additionally, they attempted to offer quantitative and data-driven methods for evaluating the efficacy of various neurosurgical approaches and techniques.

Biomarker analysis comprised 6% of the research efforts, focusing primarily on investigating potential biomarkers for early detection. These studies employed methods like gene expression analysis to assess the levels of putative biomarkers in both patients and controls. Results indicated that patients exhibited biomarker expression levels two to five times higher than those observed in the controls.

The remaining 12% of research encompassed diverse primary outcomes. Among these studies were analyses of patient paintings and explorations into the application of nanorobotics, reflecting a breadth of inquiry beyond traditional biomedical investigations.

The temporal profile of the studies included can be broken down based on their publication years as follows: In 2007, there was one study (6%); in 2013, there was one study (6%); in 2015, there was one study (6%); in 2017, there was one study (6%); in 2018, there were two studies (12%); in 2019, there was one study (6%); in 2020, there was one study (6%); in 2021, there were two studies (12%); in 2022, there were three studies (18%); in 2023, there were four studies (24%); and in 2024, there was one study (6%).

The temporal distribution reveals that the largest proportion of studies (29%) were published in the most recent year, 2023. There has been a growing trend toward using AI and ML in the field of neurology throughout the past year. The oldest study in the collection dates back to 2007.

The investigations employed various experimental models and methodologies to address research objectives efficiently. These methodologies can be broadly categorized as follows (Table 3):

**Table 3:** Summary of methodologies used in the investigations AI: artificial intelligence, ML: machine learning

Methodology	Description	Percentage of studies
Imaging Data Analysis with AI/ML Models	Utilized advanced AI/ML models like convolutional neural networks and radiomics analysis to analyze imaging data from modalities such as MRI, PET, and CT for diagnosis, prognosis, and prediction.	24%
Animal Models and In Vitro Assays	Employed animal models and in vitro assays, including techniques like immunohistochemistry and electrophysiological recordings, to evaluate nerve injury, therapeutic approaches, and drug efficacy.	24%
Analysis of Electronic Health Records and Patient Data	Applied AI/ML techniques to examine electronic health records, demographic information, and clinical laboratory tests to construct diagnostic, prognostic, and predictive models.	12%
Research on Robotic Devices for Rehabilitation	Utilized robotic systems and devices to deliver specific stimulation patterns for rehabilitating nerve damage and restoring normal function.	12%
Computational Modeling and Simulations	Employed computational techniques like ML and mathematical modeling to explore pharmacological targets and neural signaling.	6%
Analysis of Human Subject Data	Conducted data collection and analysis on human subjects, including symptoms, neuropsychological assessments, and imaging data, to develop AI/ML models.	6%

The investigations utilized a variety of AI and ML models and methodologies to address research objectives. Deep learning models were predominantly employed, constituting 41% of the methods. ML algorithms accounted for 29% of the approaches used, while radiomics analysis represented 12%. Robotic systems and devices were utilized in 12% of the investigations, with mathematical and computational modeling employed in 6% of the studies. Other AI and ML methods comprised the remaining 6% of methodologies utilized across the investigations.

Discussion

The MMAT assessment reveals heterogeneity in the quality of the research covered, encompassing a combination of high-quality, ambiguous, and low-quality studies. The methodological specifics are frequently inadequately documented, especially with participant-level and statistical elements. The limited number of mixed-methods studies usually satisfied the quality standards, although the interpretation of the combined results was not consistently evident.

The studies included in this analysis varied in quality, ranging from high quality to low quality. This indicates that the research in the field of AI and ML in neuroregenerative medicine is still developing and diverse [38]. This variation in quality emphasizes the importance of implementing more rigorous and standardized methodological approaches in future studies to improve the reliability and validity of the findings [39,40]. The lack of clarity or missing information regarding key methodological details, such as randomization, baseline comparability, and outcome assessment, highlights the need for better reporting practices in this research area [41,42].

The lack of clarity or omission of information regarding the representativeness of study samples and the appropriateness of statistical analyses suggests a need for improvement in the design and analysis of AI/ML studies in neuroregenerative medicine [43,44]. It is essential to ensure proper participant selection, consider potential confounding factors, and employ strong statistical methods in order to draw reliable conclusions from research findings. The scarcity of studies employing a mixed-methods design and the lack of obvious integration between qualitative and quantitative components indicate that the potential advantages of mixing diverse research methodologies have not been thoroughly investigated in this domain [45,46].

Advocating for the use of mixed-methods approaches, where appropriate, could enhance the depth of comprehension regarding research inquiries and yield more robust and nuanced insights. This advice is derived from the observation that only a limited number of studies included in the systematic review employed a mixed-methods design, and the incorporation of qualitative and quantitative elements was frequently ambiguous or unreported.

In the realm of AI/ML applications in neuroregeneration, employing mixed-methods approaches can provide the following benefits:

1. Triangulation and validation: The integration of quantitative data (such as model performance metrics and biomarker levels) with qualitative data (such as participant experiences and expert insights) can be used to cross-verify and validate findings. This approach improves the credibility and reliability of the results.

2. Investigation of intricate phenomena: The incorporation of qualitative methods can enable a more profound investigation of intricate phenomena, such as the interaction between AI/ML systems and neuroregeneration processes, revealing subtleties and contextual factors that may not be captured solely by quantitative data.

3. Participant perspectives: Qualitative elements, such as interviews or focus groups, can offer useful insights into the viewpoints and encounters of participants, patients, or healthcare professionals engaged in AI-/ML-driven neuroregeneration research or interventions.

4. Theory development: The utilization of mixed-methods approaches can enhance the creation and improvement of theoretical frameworks by integrating empirical quantitative evidence with detailed qualitative data, resulting in a more thorough comprehension of the fundamental mechanisms and processes.

5. Implementation and translation: Qualitative data can provide insights into the contextual factors, obstacles, and enablers linked to the implementation and translation of AI/ML technologies in neuroregeneration research or clinical settings. This information can inform strategies for effectively adopting and spreading these technologies.

Although mixed-methods approaches may not be universally applicable or possible for all investigations, promoting their utilization when fit can result in a more extensive and refined comprehension of the study inquiries in this interdisciplinary domain. Researchers can enhance the comprehensiveness and effectiveness of their discoveries in neuroregeneration by combining quantitative and qualitative data. This integration allows them to capture the intricate and multidimensional nature of AI/ML applications, leading to more robust and impactful results.

Other systematic reviews in the field of AI and ML in neuroregenerative medicine have also shown similar conclusions. For instance, Kumar (2023) conducted a systematic review of the application of ML to spinal cord injury. The review found that most studies lacked adequate information regarding their data preprocessing, feature selection, and model validation approaches. This limitation hindered the evaluation of their methodological quality [47].

Furthermore, a comprehensive analysis of the use of AI-assisted robotic rehabilitation in spinal cord injury was conducted through a systematic review. The quality of the studies included in the analysis was evaluated using the critical appraisal tools provided by the Joanna Briggs Institute (JBI) [48]. The findings revealed that most of the studies demonstrated a satisfactory level of methodological quality, although there were areas that could be enhanced, such as providing a justification for the sample size and assessing the risk of bias.

A systematic review examining the application of AI in neurorehabilitation employed the Cochrane Risk of Bias Tool for randomized controlled trials and the Risk of Bias in Non-randomized Studies of Interventions (ROBINS-I) tool for non-randomized studies [49]. The review revealed that the majority of the studies analyzed exhibited a significant or uncertain risk of bias, underscoring the necessity for more rigorous research in this domain.

To tackle this problem, it is crucial to promote the adherence of researchers to reporting guidelines, such as the Transparent Reporting of a Multivariable Prediction Model for Individual Prognosis or Diagnosis (TRIPOD) statement for predictive modeling studies or the Consolidated Standards of Reporting Trials (CONSORT) statement for clinical trials [50]. These guidelines have the objective of enhancing the transparency and comprehensiveness of reporting, facilitating precise quality evaluation, and improving the reproducibility of research outcomes.

The predominant goals were centered around the application of AI and ML in the areas of diagnosis, prognosis, and prediction, with a secondary focus on exploring robotic rehabilitation therapies. Additional primary objectives were using AI for drug discovery and gaining insights into neuro-cellular interactions.

Several additional systematic reviews in this field have also provided comparable classifications or thematic analyses of the research included. An example of this is a comprehensive analysis of the use of AI in spinal cord injury research. The analysis classified the studies it included into different areas of focus, such as diagnosis and prognosis, treatment planning, and rehabilitation [51]. The analysis revealed that the majority of the studies concentrated on diagnosing and predicting the outcomes of spinal cord injuries. This finding is consistent with our own study, where 27% of the studies investigated the diagnosis and prognosis of neurodegenerative diseases.

The analysis also discovered that the included studies attained accuracy levels between 70% and 95% for different tasks, which aligns with the performance measures mentioned in our study.

Similarly, a comprehensive analysis of the use of AI in neurodegenerative disorders found that the papers included in the review mainly concentrated on disease diagnosis, prognosis, and treatment optimization, which aligns with the themes observed in our research [52]. The systematic review also presented quantitative findings from studies examining therapeutic interventions and biomarker investigations. The review found that the studies included in their analysis reported enhancements in functional recovery scores, levels of biomarker expression, and other pertinent measures, which are consistent with the quantitative results described in our analysis.

In the study conducted by Vamathevan et al. (2019), the application of AI in drug discovery for neurorepair was examined [53]. Specifically, the researchers focused on the use of AI in identifying potential therapeutic targets and compounds for neurological disorders. They discovered that AI techniques, such as ML and virtual screening, were commonly employed in this field. These findings are consistent with the information presented in your paragraph, which states that 18% of the studies investigated the use of AI in drug discovery for neurorepair. This review focused on assessing the impact of AI in the process of discovering and developing drugs. The researchers found that the studies they analyzed showed notable improvements in different indicators of treatment effectiveness, such as the growth of neurites and functional recovery. These findings are consistent with the quantitative results we mentioned earlier in this category.

The publication chronology demonstrates an increasing amount of research in AI and ML for neurological applications in recent years, with a notable rise in 2023. This demonstrates the growing acknowledgment of AI and ML's potential in this field.

Furthermore, numerous other comprehensive evaluations in this field have also demonstrated a surge in enthusiasm for AI and ML in recent times.

The systematic review of the applications of AI in spinal cord injury research found that the majority of the included studies were published in recent years, especially from 2018 onward [51]. This corresponds with the observation that most studies (70%) in our review were published between 2018 and 2024, indicating a significant increase in research activity in this field in recent years.

Similarly, a systematic review on the utilization of AI in neurodegenerative diseases discovered that the earliest study in their review was conducted in 2008, with a notable rise in the number of published studies starting in 2018 [52]. This trend aligns with the temporal distribution observed in our research, where the earliest study dates back to 2007 and a significant number of studies have been published in recent years.

The surge in research interest and publications in the field of AI and ML in neuroregenerative medicine can be attributed to several factors. These include advancements in AI and ML techniques, particularly deep learning and neural networks, which have allowed for more precise and robust analysis of intricate neurological data [54]. Additionally, the availability of large-scale neuroimaging and clinical datasets, facilitated by collaborative efforts and data-sharing initiatives [55], has played a significant role. Furthermore, the increasing computational power and accessibility to high-performance computing resources have made it possible to train and deploy complex AI models [56]. Lastly, there is a growing recognition of the potential benefits of AI and ML in addressing challenges in neurology such as early diagnosis, personalized treatment, and drug discovery [57].

The most prevalent experimental methodologies utilized in imaging data processing were AI/ML and animal models, followed by the extraction of information from electronic records and the utilization of robotic rehabilitation devices. Several investigations also employed computational approaches and data from human subjects.

A comprehensive analysis of the use of AI in spinal cord injury research [51] revealed that the studies included in the review commonly employed techniques such as analyzing imaging data, using animal models, and conducting computational modeling and simulations. These experimental methods are consistent with the categories mentioned in our own study, which involve utilizing AI and ML models to analyze imaging data, conducting experiments on animal models, and performing computational modeling and simulations.

Moreover, a comprehensive analysis [52] of the utilization of AI in neurodegenerative diseases revealed that the studies examined frequently utilized techniques such as analysis of imaging data, analysis of electronic health records, and analysis of human subject data. These approaches align with the identified categories, namely, the utilization of AI/ML models for analyzing imaging data, electronic health records and patient data, and data obtained from human subjects.

A systematic review examined the use of robotic devices in neurorehabilitation and found that a variety of robotic systems and devices were used to provide specific interventions and enhance functional recovery [58]. This is consistent with the category of robotic devices for rehabilitation mentioned in our study.

Deep learning models were the predominant AI/ML technique, with standard ML algorithms being the next most frequently employed. Significant utilization of radiomic analysis and robotic systems was seen. Notable aspects included the use of mathematical modeling and the absence of specific reporting on AI and ML technologies.

The systematic review on the applications of AI in spinal cord injury research [51] found that the studies included in the review often used deep learning models, specifically convolutional neural networks, for tasks involving image analysis and prediction. This corresponds to the category of deep learning models mentioned in our own review.

The included research frequently utilized ML methods, such as support vector machines and random forests, for a range of prediction and classification tasks [52]. These algorithms fall into the group of ML algorithms stated in our study.

A systematic review examined the contribution of radiomics to neurological illnesses and found that multiple studies utilized radiomic features extracted from imaging data as inputs for prediction models [59]. This corresponds to the type of radiomic analysis indicated in our study.

There are several constraints associated with this systematic review. The review encompasses a combination of papers of superior, ambiguous, and inferior quality, suggesting that the current research in this domain is still developing and diverse. The absence of explicit methodological particulars in numerous studies indicates a necessity for more stringent and uniform procedures. Several studies lacked enough information regarding the representativeness of their study samples and the suitability of their statistical methods, potentially compromising the credibility of their findings. The analysis identified a dearth of studies employing combined qualitative and quantitative methodologies, which have the potential to yield more extensive and nuanced results. The review primarily focused on published papers, perhaps excluding valuable unpublished studies that could have contributed to a more comprehensive understanding of the research undertaken on this subject.

The incorporation of AI technology in neuroregeneration research gives rise to significant ethical concerns that require meticulous attention and resolution. The systematic review conducted by Bečulić et al. [60] emphasizes the significance of resolving ethical issues pertaining to privacy, data security, algorithmic bias, and responsibility when employing AI tools in medical practice and research. According to the study, a majority of the examined articles (62%) identified ethical problems as a notable risk or limitation linked to the utilization of ChatGPT. Due to the extensive training of AI models on large datasets, there is a possibility of propagating biases that exist in the training data, resulting in biased or discriminatory outputs. Moreover, the utilization of medical data for training AI models gives rise to problems regarding privacy and data security. It is imperative to establish explicit standards and laws to guarantee the responsible and ethical utilization of AI technologies. This will safeguard patient privacy and address algorithmic biases. Furthermore, it is imperative to establish transparency and accountability protocols to enable the interpretation and scrutiny of AI-assisted judgments, as well as the appropriate assignment of responsibilities. To ensure the safe and effective integration of AI in neuroregeneration research and clinical practice, it is essential to address ethical concerns by implementing strong governance structures and fostering interdisciplinary collaboration among AI developers, medical practitioners, ethicists, and policymakers.

In summary, the constraints of this systematic review emphasize the necessity for more stringent, well-documented, and comprehensive research in the utilization of AI and ML for brain regenerative medicine.

## Conclusions

Our review encompasses a wide range of objectives such as analyzing robotics for neurorehabilitation, studying neuro-cellular interactions, evaluating surgical techniques and outcomes, exploring biomarkers, and analyzing paintings by neurological patients. The study also investigates a wide variety of experimental models and approaches, including mathematical and computational modeling and simulations, analysis of paintings, and specialized techniques like Gaussian Markov random fields and transfer learning. The interest and research efforts are increasing in utilizing AI and ML techniques to progress in several facets of neuroregenerative medicine, as demonstrated by the significant rise in publications in recent years. AI and ML are extensively employed for activities such as diagnosing, forecasting, and predicting neurological disorders, with research showing encouraging performance metrics. Robotics and targeted stimulation devices equipped with AI capabilities are becoming more effective tools for neurorehabilitation and facilitating functional recovery. The review also emphasizes the potential of AI-driven drug discovery and optimization to identify new therapeutic candidates for brain regenerative treatments. AI's potential extends to neurorehabilitation and drug discovery. Further research is crucial to address current limitations.
